# Response to “Reassessing the death risk related to probiotics in critically ill patients”

**DOI:** 10.1186/s13054-017-1618-0

**Published:** 2017-02-27

**Authors:** William Manzanares, Paul E. Wischmeyer

**Affiliations:** 1 Department of Critical Care, Intensive Care Unit, Hospital de Clínicas (University Hospital). Faculty of Medicine, Universidad de la República (UdelaR), Italia Ave. 14th Floor, 11.600, Montevideo, Uruguay; 2 Department of Anesthesiology and Surgery, Duke University School of Medicine, Duke Clinical Research Institute, 2400 Pratt Street, Office: NP 7060, Durham, NC 27705 USA

We would like to thank Dr. Maraolo for his valuable and careful analysis [1] of the data of our recently published systematic review and meta-analysis on probiotic and synbiotic therapy in the critically ill [2]. As Dr. Maraolo has observed we have made an error in the calculation of the pooled risk ratio (RR) and 95% confidence interval (CI) for the effect of probiotics on hospital mortality. When we abstracted mortality data from the Besselink et al. [3] study we included correct data in both arms (24 of 152 and 9 of 144 patients in the probiotic and placebo groups, respectively). Nonetheless, we made a mistake creating the forest plot. Please, accept our sincere apologies.

Currently, after including the correct data from the Besselink et al. study using the random effect model in the software RevMan 5.3 (Cochrane IMS, Oxford, UK), we found that the revised effect of probiotics and synbiotics therapy on overall mortality is 1.02 (95% CI 0.85,1.22; *p* = 0.83, I^2^ = 0%; Fig. 1). Notwithstanding, at this point we respectfully disagree with Dr. Maraolo. Certainly, after reassessing the RR this new result does not change the direction of the effect against the use of probiotics in the critically ill. Our previous data showed that the RR was 0.98 with a CI similar to the present one (0.85, 1.22). Moreover, the *p* value was 0.83 and we cannot thus affirm that a trend against probiotics on mortality exists, as we defined trend with a *p* value <0.10. So far, clinical trials evaluating the effects of probiotics (excluding *Saccharomyces boulardii*, which should not be considered as a probiotic in the critical care setting) [4] in different ICU patient populations have documented safety and clinical benefits, as we recently demonstrated in our systematic review.

**Fig. 1:**
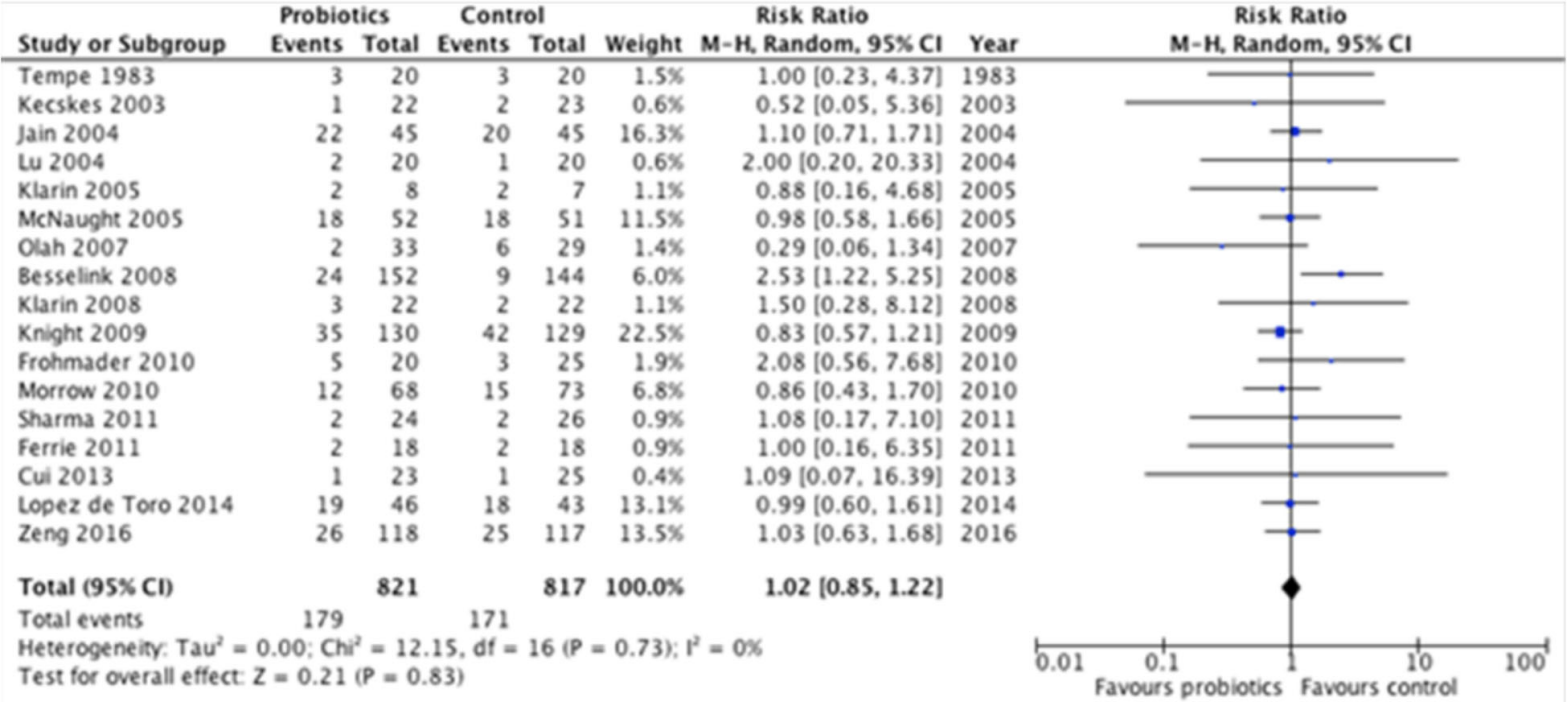
Effect of Probiotics and Synbiotics Therapy on Hospital Mortality

Having said that, the conclusion of our meta-analysis remains unchanged. According to our findings probiotic therapy may be associated with a significant reduction in overall new infections, including new episodes of ventilator-associated pneumonia. However, no benefits in terms of reduction in mortality or another relevant clinical outcome for critically ill patients have been pointed out.

## Abbreviations

CI: Confidence interval
RR: Risk ratio.
